# Characterisation of Ex Vivo Liver Thermal Properties for Electromagnetic-Based Hyperthermic Therapies

**DOI:** 10.3390/s20103004

**Published:** 2020-05-25

**Authors:** Nuno P. Silva, Anna Bottiglieri, Raquel C. Conceição, Martin O’Halloran, Laura Farina

**Affiliations:** 1 Translational Medical Device Lab, National University of Ireland Galway, H91 TK33 Galway, Ireland; n.silva1@nuigalway.ie (N.P.S.); a.bottiglieri1@nuigalway.ie (A.B.); martin.ohalloran@nuigalway.ie (M.O.); 2 Faculdade de Ciências da Universidade de Lisboa, 1749-016 Lisbon, Portugal; 3 Instituto de Biofísica e Engenharia Biomédica, Faculdade de Ciências Universidade de Lisboa, 1749-016 Lisbon, Portugal; rcconceicao@fc.ul.pt; 4 CÚRAM, SFI Research Centre for Medical Devices, National University of Ireland Galway, H91 W2TY Galway, Ireland

**Keywords:** electromagnetic-based therapies, thermal properties, hyperthermia, thermal ablation, density, water loss

## Abstract

Electromagnetic-based hyperthermic therapies induce a controlled increase of temperature in a specific tissue target in order to increase the tissue perfusion or metabolism, or even to induce cell necrosis. These therapies require accurate knowledge of dielectric and thermal properties to optimise treatment plans. While dielectric properties have been well investigated, only a few studies have been conducted with the aim of understanding the changes of thermal properties as a function of temperature; i.e., thermal conductivity, volumetric heat capacity and thermal diffusivity. In this study, we experimentally investigate the thermal properties of ex vivo ovine liver in the hyperthermic temperature range, from 25 °C to 97 °C. A significant increase in thermal properties is observed only above 90 °C. An analytical model is developed to model the thermal properties as a function of temperature. Thermal properties are also investigated during the natural cooling of the heated tissue. A reversible phenomenon of the thermal properties is observed; during the cooling, thermal properties followed the same behaviour observed in the heating process. Additionally, tissue density and water content are evaluated at different temperatures. Density does not change with temperature; mass and volume losses change proportionally due to water vaporisation. A 30% water loss was observed above 90 °C.

## 1. Introduction

Electromagnetic-based thermal therapies are currently widely adopted in clinical practice to treat cancerous diseases. The interaction between an electromagnetic field and the target biological tissue induces a localised and controlled increase in temperature. The induced increase of temperature depends on the purpose and characteristic of the hyperthermic treatment: hyperthermia raises the temperature in the target area up to 40–45 °C in order to cause an increase of the cellular metabolism, perfusion and oxygenation, while thermal ablation increases the temperature of the target above 55 °C to induce an irreversible damage to the tissue [1,2,3,4]. Dielectric and thermal properties play an important role in the electromagnetic and thermal phenomena occurring in the tissue targeted by an hyperthermic treatment. The accurate knowledge of these properties can help predict the electromagnetic energy deposition and the distribution of the temperature increase in the tissue, in order to optimise treatment plans [4,5,6,7]. While dielectric properties have been widely studied [8,9,10,11,12,13,14,15,16,17,18,19,20], less systematised investigations have been conducted to characterise the thermal properties of biological tissues and their changes with temperature.

Thermal properties, such as thermal conductivity, volumetric heat capacity and thermal diffusivity, influence the temperature increase rate and the heat distribution in biological tissues [4]. Thermal conductivity, *k* [W m^−1^ K^−1^], measures the ability of the tissue to conduct heat, while thermal diffusivity, *D* [m^2^ s^−1^], describes how quickly a material reacts to a variation in temperature. Volumetric heat capacity, *C_v_* [J m^−3^ K^−1^], relates to the quantity of heat required to raise one degree of temperature of the tissue, per unit of volume. These thermal properties are interrelated:

(1) $$D=\frac{k}{C_{v}}$$

Over the last 30 years, studies have been conducted to investigate temperature-dependent thermal properties at hyperthermic temperatures (above 40 °C), investigating several tissues, using different measurement methodologies, and leading to different results [21,22,23,24,25,26,27,28]. It is important to acknowledge that most of these studies have investigated thermal properties in ex vivo biological tissues in steady state heat transfer conditions; i.e., neglecting the influence of blood perfusion. An “effective” thermal conductivity and an “effective” thermal diffusivity should be considered, instead, to account for the contribution of perfusion to the steady state heat transfer in vivo [29].

A self-heated thermistor inserted into the tissue was used by Valvano et al. in [21] to determine the thermal conductivity and diffusivity of different ex vivo biological tissues. This method measures the baseline tissue temperature; then, electrical power is applied to the thermistor in order to heat it. Thermal conductivity and diffusivity are derived from the difference in temperature recorded. The authors measured both the thermal conductivity and thermal diffusivity of different ex vivo biological tissues from different species in a temperature range between 3 °C and 45 °C. Differences between tissues, species, and even within tissues from the same donor were reported. A similar measurement method was used to determine the thermal conductivity of freshly excised porcine myocardium, at temperatures between 25 °C and 76 °C, by Bhavaraju et al. [22]. A decrease of thermal conductivity with the temperature increase was reported and justified by the authors, with significant water loss occurring at temperatures above 50 °C. The same measurement technique was used by Choi et al. [27] to measure the thermal conductivity and apparent specific heat capacity of ex vivo human and porcine liver for a range of temperatures between 20 °C and 85 °C. This study reports an increase of thermal conductivity of approximately 12% in the range of investigated temperatures and an increase of specific heat capacity up to approximately 70 °C, decreasing after that. The authors correlated the changes of thermal properties observed with protein denaturation and water loss.

A transient hot-wired technique, which applies a fixed potential difference across the probe and records differences in temperature, was used by Bhattacharya et al. [23] to measure the thermal conductivity of ex vivo bovine liver tissue in a temperature range from 25 °C to 90 °C. In the study, it was observed that thermal conductivity varies linearly with temperature.

A study on the dependency of specific heat capacity on temperature was performed by Haemmerich et al. [24] on ex vivo liver in a temperature range from 20 °C to 85 °C. The sample was uniformly heated between two electrodes. The specific heat capacity was observed to increase for temperatures above 65 °C; this increase was correlated to the tissue water loss due to heating.

Recent studies used a commercial thermal analyser device to investigate thermal properties (thermal conductivity, volumetric heat capacity and thermal diffusivity) of ex vivo liver in a temperature range from 20 °C to 90 °C (Guntur et al. [26]) and from 21 °C to 113 °C (Lopresto et al. [28]). Guntur et al. observed that thermal properties change with temperature following an asymmetric quasi-parabolic convex downward curve with a minimum at body temperature and increasing afterwards. In [26], measurements were also taken after tissue reached 90 °C while the tissue was left to cool. The authors observed that thermal properties remained unchanged, pointing out the irreversibility of the variations in thermal properties observed at 90 °C. In contrast, Lopresto et al. observed that thermal properties do not vary with temperature until 90 °C; above this temperature, thermal properties increase exponentially until the water phase transition process is completed.

The increase of temperature alters the characteristics of the tissue. Tissue appearance changes above 50–60 °C due to coagulation processes, and above 100 °C, the tissue carbonises due to tissue desiccation induced by the vaporisation of the water content [30,31]. Additionally, shrinkage of the tissue was reported to occur at ablative temperatures [28,32]. The water vaporisation process appears to dramatically influence the thermal properties of the tissue; Lopresto et al. [28] observed a huge drop of thermal properties for temperatures above 100 °C.

The proposed study aims to better understand how thermal properties change during and after hyperthermic electromagnetic-based therapies. In this study, we characterise the thermal properties of ex vivo ovine liver as a function of the temperature increase for temperatures used in hyperthermia and/or thermal ablation up to 97 °C. We validate the methodology presented in [28] and compare the results obtained in two different animal models. Then, a set of measurements is conducted during the cooling of the tissue to observe the reversibility of the changes in thermal properties observed above 90 °C. Changes in tissue density and water content with temperature are investigated during the experimental study and correlated with the variation of thermal properties. To the best of our knowledge, this is the first study to also address the influence of the variation in the density and water content of tissue with temperature.

## 2. Materials and Methods

**Figure 1:**
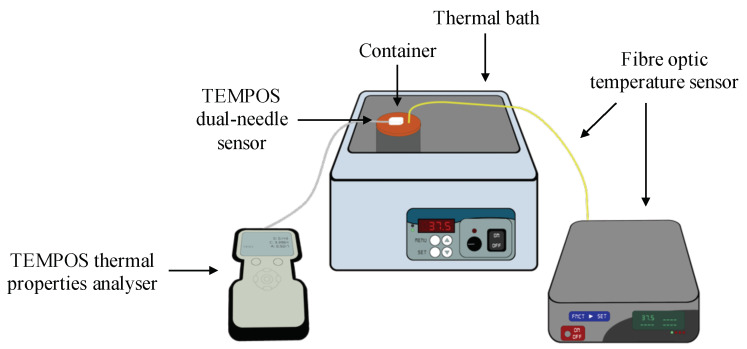
Experimental setup: TEMPOS thermal properties analyser, metallic container, thermal bath and fibre optic temperature sensor [33].

The experimental set-up used to investigate the thermal properties of ex vivo ovine liver as a function of temperature is illustrated in Figure 1. Thermal properties measurements were conducted with a commercial analyser (TEMPOS, Meter Group, Inc., Pullman, WA, USA, accuracy: 10%). The device was equipped with different sensors which were adapted to measure the thermal properties of different materials, depending on their composition (e.g., solid, liquid or moist) and expected thermal behaviour (e.g., conductor or insulator). The most suitable sensor to measure the thermal properties of biological samples is the SH-3 dual-needle sensor, as the sensor measures thermal conductivity, volumetric heat capacity and thermal diffusivity in non-liquid materials.

**Figure 2:**
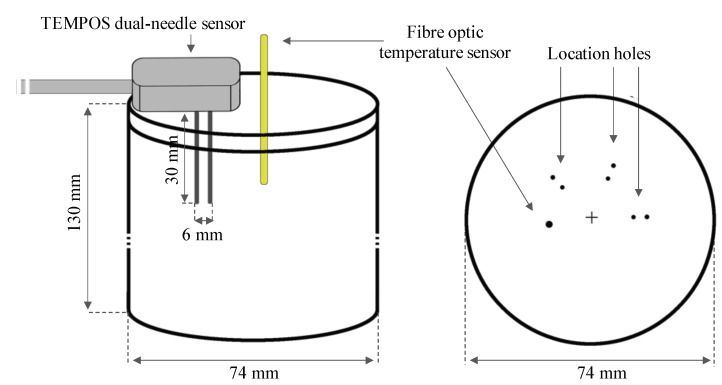
Sketch of the container used to hold the samples, with the related dimensions. **Left**: The dual-needle SH-3 sensor from the TEMPOS thermal properties analyser is sketched. The sensor was placed at least 15 mm from the border of the container, according to the manufacturer’s specification. A fibre optic temperature sensor used to monitor the temperature during the experiment is also shown. **Right**: The container’s lid is sketched together with the holes dedicated to the insertion of the SH-3 sensor and the fibre optic temperature sensor [33].

A water thermal bath (Fisher Scientific, Isotemp^®^, Waltham, MA, USA, resolution: 0.1 °C) was used to control the temperature during measurements. A metallic container was used to place the tissue samples inside the thermal bath and to prevent the contamination of the samples with water; the conductive material of the container eased the heating of the sample. The temperature of the tissue was also monitored with a fibre optic temperature sensor (Neoptix^TM^, Inc. Quebec, QC, Canada, resolution: 0.1 °C) inserted into the sample under test. The lid of the metallic container was drilled with three different sets of dedicated holes for the insertion of the dual-needle sensor and one dedicated hole for the fibre optic temperature sensor, as depicted in Figure 2.

A precision balance (Ohaus^®^, Greifensee, Switzerland, resolution: 0.1 g) was used to estimate the water losses which occurred in tissue samples due to the temperature increase. Lastly, a ruler was used to evaluate the samples’ dimensions.

### 2.1. Thermal Property Measurement Method

The thermal property measurements were conducted in ex vivo biological tissues using the SH-3 dual-needle sensor, made by two parallel needles which were 30 mm long, 1.3 mm in diameter and *r* = 6 mm spaced (see Figure 2).

The measurement mechanism of the SH-3 sensor consists of the application of a determined quantity of heat, *q*, to one of the two parallel needles (the heating needle) for 30 s, *t_h_*. The heat is transferred from the heating needle to the tissue under testing. The temperature change is recorded by the other needle (monitoring needle) for 90 s. The initial temperature, *T_0_*, recorded at the beginning of the heating period, is then subtracted from the temperature value recorded from the monitoring needle, *T*, to determine the increase of temperature in time: $\Delta T=T-T_{0}$. Thermal conductivity, *k*, and thermal diffusivity, *D*, are derived by a least square method (Equations (2) and (3)) in order to find the best values that minimise the square differences between measured and modelled temperatures:

(2) $$\Delta T=\left[\frac{q}{4\pi k}\right]E_{i}\left[\frac{-r^{2}}{4Dt}\right],$$

(3) $$\Delta T=\left[\frac{q}{\pi k}\right]\left\{E_{i}\left[\frac{-r^{2}}{4D(t-t_{h})}\right]-E_{i}\left[\frac{-r^{2}}{4Dt}\right]\right\}.$$

Equations (2) and (3) depend on the measurements performed during the heating and cooling periods, respectively. $E_{i}\left[x\right]$ corresponds to the exponential integral function, which for small arguments, *x*, approximates the function to the sum of the Euler–Mascheroni constant (γ = 0.5772) to the natural logarithm of the argument (ln(x)). Then, the volumetric heat capacity is derived from the relation between the thermal properties (see Equation (1)). An estimate error value, $S_{yx}$ [°C], indicating how well the Equations (2) and (3) fit the measured data, is reported by the device for each measurement.

### 2.2. Experimental Protocol

A total of 22 ex vivo ovine livers were used in this study. Twenty-one livers were purchased from a local butcher, who received them on the same day from the abattoir. After their excision from the animal, the livers were stored in a fridge between 1–2 °C in a sealed vacuum container in order to minimise their dehydration. When purchased from the butcher, the tissues were kept in the sealed container at room temperature. One liver was obtained directly from the animal, freshly excised, and measurements were conducted up to 3 h after excision. A total of *n* = 24 samples were obtained from the 22 livers.

Samples were cut to fit inside the metallic container, identifying the most homogeneous part of each sample to avoid variation in the tissue density—i.e., in-homogeneity—which might contaminate the results (e.g., large vessels). The samples were cut to satisfy the minimum requirements for the dimensions of the sample as specified by the manufacturer of the TEMPOS device [34]: at least 15 mm of homogeneous tissue should surround the sensor in any parallel direction to ensure an optimal measurement standard. Then, the metallic container was covered with the lid with dedicated holes for the insertion of the TEMPOS dual-needle sensor and the fibre optic temperature sensor, as depicted in Figure 2.

The fibre optic temperature sensor was inserted into the sample, by approximately 30 mm, and the dual-needle sensor was introduced in one of the three dedicated locations through the lid. The metallic container was placed into the thermal water bath to reach the desired temperature and to keep the temperature of the sample constant during the whole experimental procedure; in each set of measurements, a specific temperature was set at the thermal bath. Samples were kept inside the thermal bath to reach the desired temperature under 3 h, depending on the target temperature. Then, thermal measurements were conducted under two experimental conditions:

1. Measurements were conducted to investigate the thermal properties of liver tissue as a function of the increase of temperature (*n* = 21). For each sample, the thermal bath was set to specific temperatures, and the following were tested: 25 °C, 27 °C, 36 °C, 40 °C, 44 °C, 49 °C, 55 °C, 60 °C, 70 °C, 80 °C, 92 °C and 97 °C. Measurements were performed only when the whole sample reached thermal equilibrium with the thermal bath temperature. In each sample, measurements were taken in three different locations. Thermal property measurements were repeated three times in each location with a time interval of 10 min. When the sensor was moved from one location to another, it was left in place 15 min before taking the next measurement. These time intervals guaranteed thermal equilibrium between the sensor and the tissue.
2. Measurements were conducted to investigate the thermal properties of liver tissue as a function of the decrease of temperature (*n* = 3). The sample was placed in the thermal bath set at 90 °C (*n* = 2) or 95 °C (*n* = 1). After the sample reached the desired temperature, the thermal bath was turned off and single measurements were conducted always in the same location every 30 min, until the sample reached body temperature (37 °C).

Before and after any experimental procedure, the TEMPOS dual-needle sensor was verified according to the manufacturer’s instructions. In all experiments, the measurements were performed using a TEMPOS dual-needle sensor in agreement with the required manufacturer conditions [34].

Measurements were conducted to evaluate the density of the samples and their water content as a function of temperature. For experiments above 45 °C, samples were weighed before (at room temperature) and after the experiment, in order to determine the water loss of the sample due to the increase of temperature. Moreover, a portion of each liver was cut in an approximated cubic-shape. The cubic-shaped sample was weighed on a precision scale to measure the mass. The volume of the sample was estimated by measuring all edges of the cube with a ruler: height, length and width. The volume of the sample was calculated by the average height, length and width. Volume calculations were performed three times, changing the cube orientation with respect to the bench. The density of the sample was calculated by the average of the three measured volumes divided by the mass weighed with a precision scale [33]. This method was used to estimate the tissue density at room temperature and after the heating process.

### 2.3. Measurement Uncertainty

The associated density uncertainty was determined by the propagation of the uncertainties concerning the volume and mass measurements conducted using the ruler and the precision scale, respectively [35].

The uncertainties of the thermal property measurements were calculated taking into account a guideline for the evaluation and the expression of the uncertainty of measurement results [36]. To achieve this, different sources of error were identified and quantified in order to provide the combined uncertainty, which represents the estimated standard deviation of the results. The first source of error is correlated to the measurement results obtained for each thermal property. The repeatability and reproducibility of those results correspond to the standard deviation of the mean (SDM), giving information about the precision of the mean obtained during measurement campaigns, which is determined by

(4) $$SDM=\sqrt{\frac{\sum_{n}^{i=1}{\left(x_{i}-\bar{x}\right)}^{2}}{n(n-1)}},$$

where $x_{i}$ is the *i*th data point and $\bar{x}$ is the average value obtained from the total *n* measurements.

The uncertainty was evaluated considering the values obtained for each thermal property using Equation (4) and the accuracy specified by the manufacturer of the device [34]. The instrument uncertainty was divided by $\sqrt{3}$ assuming its rectangular/uniform distribution. The combined uncertainty was then calculated by the square root of the arithmetic sum of the square of each uncertainty. Afterwards, the expanded uncertainty was calculated by multiplying the combined uncertainty with the coverage factor, K=2. This defines a confident level interval of 95% in which a measurement is likely to occur, and it defines the uncertainty of each thermal property measurement.

### 2.4. Thermal Property Modelling with Temperature

A model was fitted for each thermal property as a function of temperature, using the data obtained in our experimental measurements together with literature data [28] obtained with an experimental setup similar to the one presented in this study. The analytical function adopted characterises the changes for each thermal property (*Y*)—i.e., thermal conductivity, volumetric heat capacity and thermal diffusivity—as a function of temperature (*T*) in a range from 20 °C to 99 °C:

(5) $$Y\left(T\right)=a+be^{cT},$$

where *a*, *b* and *c* are the regression coefficients that best fit the data. The coefficient of determination ($R^{2}$) was also evaluated as a measure of how well the model replicates the data.

## 3. Results and Discussion

### 3.1. Thermal Properties: Temperature Increase Measurements

Thermal properties of ex vivo ovine liver samples were measured at different temperatures, from room to ablative temperatures: 25 °C, 27 °C, 36 °C, 40 °C, 44 °C, 49 °C, 55 °C, 60 °C, 70 °C, 80 °C, 92 °C and 97 °C. Three measurements in three different locations in each sample (with a total of nine measurements per sample) were conducted on *n* = 21 samples. In Table 1, the average of each thermal property measured at a specific temperature is reported independently of the location of the sensor in the sample. Additionally, the related uncertainty of the average results, calculated as explained in Section 2.3, is presented.

No difference was observed between the results obtained from the samples collected from the local butcher and those obtained from the freshly excised samples; this supports the effectiveness of the storage methodology adopted.

As reported in Table 1, the thermal properties of ex vivo ovine liver are approximately constant with increasing temperature. Sizable changes in the thermal properties are observed for temperatures above 92 °C. Increases of about two times for thermal conductivity and of about 1.5 times for volumetric heat capacity and thermal diffusivity are observed between 92 °C and 97 °C. The uncertainty of the measurements is about 10%, in compliance with the accuracy of the device: 11%–12% for thermal conductivity and volumetric heat capacity, and 11%–14% for thermal diffusivity. Additionally, the uncertainty increases for higher temperatures; at 97 °C, 15% uncertainty is observed for thermal conductivity, 18% for volumetric heat capacity and 22% for thermal diffusivity.

**Figure 3:**
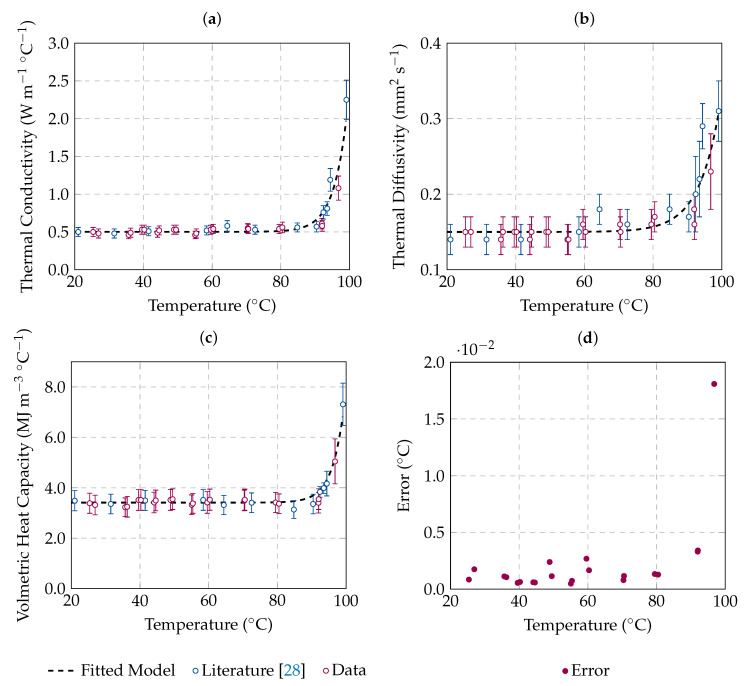
Thermal properties of ex vivo ovine liver as a function of the increasing temperature: (**a**) thermal conductivity, (**b**) thermal diffusivity and (**c**) volumetric heat capacity. Average values are reported with the associated uncertainty (error bars). Experimental data (red) as well as literature data (blue) are shown. The best fit model is also presented by a dashed line. The estimated error of how well the least square model fits the data is reported in (**d**).

The data reported in Table 1 are graphed in Figure 3 as a function of temperature as it increases. The average values obtained for each thermal property, with the associated uncertainty, are shown. Our experimental results are reported in the figure together with literature data reported in [28], where an experimental setup similar to the one implemented in this study was adopted to characterise the thermal properties of ex vivo bovine liver. In [28], the average values for each thermal property as a function of temperature and the uncertainty associated with the measurements were reported. Additionally, the best fit model obtained following the numerical regression function presented in Equation (5) in Section 2.4 is reported in Figure 3 for each thermal property. The model was computed using both the data obtained in our study—Table 1—and the literature data [28]. Since no significant differences were observed among species (ovine and bovine), the data were pooled together to strengthen the model. The regression coefficients of the model, as well as the $R^{2}$ coefficient for each thermal property, are reported in Table 2.

As observed in Figure 3, thermal properties exponentially increase when temperatures above 90 °C are reached. Such findings can be related to the water vaporisation process that occurs close to 100 °C at sea level (1 atm) . In addition, it is observed that the thermal properties obtained in this study are in agreement with the values reported in the literature [28], despite the different rate of heating (e.g., to reach 90 °C, 3 h were needed in this study, while 14 h were required in [28]). The model fits the data with a R-squared coefficient higher than 0.9 for thermal conductivity ($R^{2}$ = 0.93) and volumetric heat capacity ($R^{2}$ = 0.95), while the thermal diffusivity model shows an R-squared coefficient of 0.81. In all cases, the model is able to predict the data obtained in the experimental procedure. It is worth noting that literature data were measured in ex vivo bovine liver, while ex vivo ovine liver was used in this study. Both measured and literature data show similar results for the same tissue type from different species, in contrast to the observations by Valvano et al. [21].

The error obtained by the instrument and presented in Figure 3d tells us how well the measurement algorithm of Equations (2) and (3) fits the measured data. Higher error values are mainly associated with fluctuations in temperature; i.e., temperature not constant during measurement procedure. At higher temperatures (above 90 °C), the water state transition occurs; approaching 100 °C, the vaporisation process impacts our measurements, causing fluctuations in temperature and increasing the error associated to measurements, as observed in Figure 3d. Additionally, the increase of the uncertainty observed above 90 °C is in agreement with the increase of the error obtained by the instrument.

### 3.2. Thermal Properties: Temperature Natural Decrease Measurements

**Figure 4:**
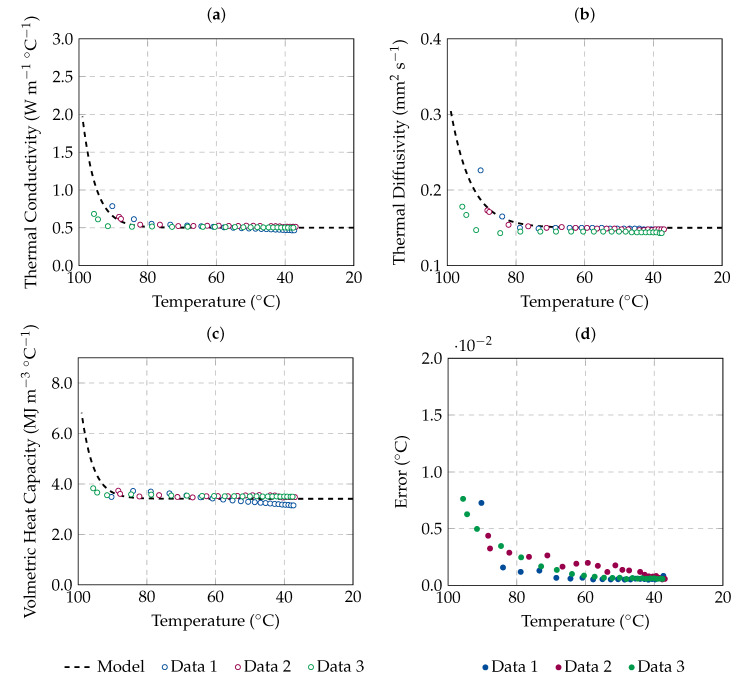
Thermal properties of ex vivo ovine liver as a function of the decreasing temperature: (**a**) thermal conductivity, (**b**) thermal diffusivity and (**c**) volumetric heat capacity. Raw data measured during the cooling of each sample are reported; their nominal associated uncertainty is 10%, which corresponds to the device accuracy. The best fit model described in Table 2 is also reported by a dashedline. The estimated error of how well the least square model fits the data is reported in (**d**).

Measurements were conducted during the cooling of *n* = 3 samples from a starting temperature of 90 °C (*n* = 2) and 95 °C (*n* = 1) to body temperature (37 °C). The aim of these measurements is to understand the reversibility of the changes in thermal properties. The samples were kept for 3 h in a thermal bath to reach 90–95 °C. Afterwards, the thermal bath was turned off to allow the cooling of the samples. The thermal properties of the samples under testing were measured every 30 min until body temperature (37 °C) was reached, which took approximately 10 h. The results are shown in Figure 4. In each graph, the fit analytical model presented in the previous section (Table 2) is also reported.

As shown in Figure 4, values of thermal properties higher than the baseline are observed for temperatures above 90 °C; during the cooling process, the thermal properties values decrease following the trend suggested by the analytical model, without showing any hysteresis phenomena. The results obtained during the tissue cooling are in agreement with the results reported in Table 1 and Figure 3. Thus, it is possible to observe that the changes in thermal properties with high temperatures are reversible when complete vaporisation of the tissue water content does not occur; i.e., when a temperature of 100 °C is not reached. The tissue thermal properties return to their basal values during the cooling. In contrast, Guntur et al. [26] observed that changes which occurred at 90 °C in the same biological tissue were irreversible. In [26], the samples were embedded in phosphate-buffered saline (PBS) before the experiment, and it is unknown how long the samples were kept at a temperature of 90 °C. Thus, the tissue could have become completely desiccated, and a direct comparison between the two set of data is not possible.

Additionally, as observed in Figure 4d, the estimated error given by the instrument is high for temperatures above 85 °C. This increase in the estimated error with temperatures closer to 100 °C was also observed during the measurements at constant temperature (see Figure 3d). At temperatures close to 100 °C, the water vaporisation process causes temperature fluctuations, affecting the ability of the algorithm to fit the measured data.

### 3.3. Water Loss and Density Evaluation

Fifteen of the *n* = 21 samples used in the experimental study of Section 3.1 were weighed before and after the experiment to determine the water loss. Mass measurements were conducted in samples that reached temperatures above 45 °C. The results show an increase of water loss with the increase of temperature. Below 60 °C (i.e., at temperatures of 45, 50, 55 and 60 °C) an average water loss of 12 ± 3% is observed; while at 70 °C, an average water loss of 21 ± 2% is recorded. Higher percentages of water loss are measured at temperatures higher than 90 °C, close to the water transition phase (100 °C). At 90 °C, the water loss percentage is approximately 28 ± 1%, while at 95 °C (one sample), 34% of water loss is observed.

Density measurements were conducted on the total of 22 ex vivo ovine livers at room temperature: the average density obtained is 1128 ± 75 kg m^−3^. The density reported in the literature for liver is 1079 ± 53 kg m^−3^ [37]. The density measured in this study at room temperature agreed with the literature data. The density obtained after the heating process showed density values in the same range as those obtained at room temperature: e.g., 1028 ± 58 kg m^−3^ at 60 °C; 1031 ± 85 kg m^−3^ at 90 °C; and 1104 ± 78 kg m^−3^ at 95 °C.

A proportional percentage loss in mass and volume was observed, explaining the similarity observed in the density of the samples before and after the increase of temperature: e.g., we observed comparable percentage losses in mass (24%) and in volume (23%) at 90 °C. Since density is the ratio between mass and volume, such similar losses imply no significant differences in density with the increase of temperature; the mass loss is due to the water vaporisation process and it is compensated by the volume loss. Moreover, the loss in volume observed is comparable to the shrinkage values reported in [5]; Rossmann et al. observed that, between 85 °C and 95 °C, the shrinkage of ex vivo liver tissue varied between 17.1% and 24.6%.

Thus, we observe no variation of the tissue density with increasing temperature, while an increase of the water loss with temperature is observed along an increase of the thermal properties. This correlates with the increase in water thermal conductivity that can be observed close to 100 °C; previous studies observed that the thermal properties of water increase with temperature, until the phase transition is complete [27,38]. A similar behaviour can be observed for ex vivo liver (see Table 1 and Figure 3).

## 4. Conclusions

In this study, a thorough characterisation of thermal properties of ex vivo liver was conducted, in steady state heat transfer conditions, to investigate the thermal property changes as a function of the temperature increase in the range of the hyperthermic therapeutic application. No changes in thermal properties are observed in the range of hyperthermia temperatures (40–45 °C) and for coagulative temperatures (50–60 °C). At higher temperatures (above 90 °C), approaching the water vaporisation process (at 100 °C), an increase of the thermal property values was recorded. These results are in agreement with the most recent results in the literature. Moreover, the changes in thermal properties observed above 90 °C were shown to be reversible: the tissue was allowed to cool down and reach body temperature, and the thermal properties decreased following a trend similar to that observed during heating. Finally, an analytical model is proposed to represent the trend of the thermal properties of ex vivo liver as a function of temperature up to 100 °C.

Additionally, density variations and changes in water content were evaluated as a function of temperature and correlated with the thermal properties changes. No variations in density were observed; with the increase of temperature, the water vaporises, and the tissue proportionally shrinks. However, a decrease in the water content can be inferred due to the observed mass loss. The tissue water content indicates that water has a key role in influencing the thermal properties of the biological tissue. The thermal properties of liver tissue exhibited a behaviour similar to that of water; the thermal properties of water increase with temperature (until the transition phase is reached), similar to our observations of the thermal properties of liver. Moreover, we observed that if the water phase change does not occur—i.e., if complete dehydration of the tissue is not reached—changes in tissue properties are reversible.

Further studies are needed in order to better understand the complex mechanisms associated with the changes of thermal properties with temperature in steady state heat transfer conditions and in the presence of perfusion. We have observed in this study that as long as the water content is dominant, it leads the thermal behaviour; thus, the dehydration linked to the excision may not significantly impact the property values. However, the presence of flow impacts the quantity and rate of heating and in turn the thermal behaviour of the tissue [29]. Biological tissues of interest, other than liver, should be investigated as a function of increasing temperature. The characterisation of thermal properties for other biological tissues would be a step forward in the development of better treatment models for hyperthermic therapies. Additionally, it would be important to replicate the heating study at higher rates of temperature changes, as well as the cooling study, to observe the reversibility of the thermal properties before and after the water transition from liquid to gas (100 °C).

## Figures and Tables

**Table 1:** Thermal properties (i.e., thermal conductivity, *k*, volumetric heat capacity, $C_{v}$, and thermal diffusivity, *D*) for each ex vivo ovine liver sample (*n*) at different increasing temperatures (average values, avg). The associated uncertainty of each thermal property is given by $u_{e}$.

*n*	*T* [°C]		*k* [W m^−1^ K^−1^]		$C_{v}$ [MJ m^−3^ K^−1^]		*D* [mm^2^ s^−1^]	
	avg	$u_{e}$	avg	$u_{e}$	avg	$u_{e}$	avg	$u_{e}$
**1**	25.35	0.10	0.50	0.06	3.39	0.40	0.15	0.02
**2**	26.93	0.76	0.48	0.06	3.32	0.39	0.15	0.02
**3**	35.68	0.03	0.46	0.05	3.23	0.37	0.14	0.02
**4**	36.27	0.12	0.49	0.06	3.25	0.41	0.15	0.02
**5**	39.55	0.11	0.53	0.06	3.52	0.41	0.15	0.02
**6**	40.24	0.07	0.53	0.06	3.52	0.41	0.15	0.02
**7**	44.11	0.33	0.49	0.06	3.42	0.41	0.14	0.02
**8**	44.58	0.09	0.52	0.06	3.50	0.41	0.15	0.02
**9**	48.87	0.22	0.53	0.06	3.52	0.41	0.15	0.02
**10**	49.49	0.18	0.53	0.06	3.55	0.41	0.15	0.02
**11**	55.05	0.06	0.46	0.05	3.33	0.39	0.14	0.02
**12**	55.37	0.14	0.48	0.06	3.38	0.39	0.14	0.02
**13**	59.59	0.21	0.53	0.06	3.42	0.43	0.16	0.02
**14**	60.34	0.17	0.54	0.06	3.53	0.42	0.15	0.02
**15**	70.37	0.26	0.55	0.06	3.50	0.41	0.16	0.02
**16**	70.52	0.46	0.54	0.06	3.53	0.42	0.15	0.02
**17**	79.49	0.44	0.54	0.06	3.42	0.40	0.16	0.02
**18**	80.46	0.25	0.56	0.07	3.37	0.39	0.17	0.02
**19**	91.97	0.52	0.61	0.07	3.41	0.41	0.18	0.02
**20**	92.03	0.16	0.58	0.07	3.55	0.41	0.16	0.02
**21**	96.79	0.15	1.08	0.16	5.05	0.89	0.23	0.05

**Table 2:** Regression coefficients and R-squared of fit function modelling each thermal property with temperature.

Thermal Property	*a*	*b*	*c*	$R^{2}$
Thermal conductivity, *k* [W m^−1^ K^−1^]	0.502	1.447 $\times \;10^{-11}$	0.256	0.93
Volumetric heat capacity, $C_{v}$ [MJ m^−3^ K^−1^]	3.415	1.278 $\times \;10^{-12}$	0.289	0.95
Thermal diffusivity, *D* [mm^2^ s^−1^]	0.150	1.379 $\times \;10^{-8}$	0.164	0.81
